# Prostate cancer as a first and second cancer: effect of family history

**DOI:** 10.1038/sj.bjc.6605263

**Published:** 2009-08-18

**Authors:** H Zhang, J L Bermejo, J Sundquist, K Hemminki

**Affiliations:** 1 Division of Molecular Genetic Epidemiology, German Cancer Research Center (DKFZ), Heidelberg 69120, Germany; 2 Institute of Medical Biometry and Informatics, University Hospital Heidelberg, Heidelberg 69120, Germany; 3 Center for Primary Health Care Research, Lund University, Malmö 20502, Sweden; 4 Center for Family and Community Medicine, Karolinska Institute, Alfred Nobels alle 12, Huddinge 14183, Sweden

**Keywords:** familial risk, prostate cancer, second primary malignancy

## Abstract

**Background:**

Diagnosis with prostate cancer has been reported to increase the risk of subsequent tumours. However, specific data on individuals with a parental history are not available so far.

**Methods:**

On the basis of the nationwide Swedish Family-Cancer Database including 18,207 primary invasive prostate cancers, standardised incidence ratios (SIRs) were used to estimate the relative risks of subsequent tumours after prostate cancer in the general population and among individuals with a parental history of cancer.

**Results:**

A significantly increased SIR of colorectal cancer was found among prostate cancer patients with a parental history of colorectal cancer (2.26, 11 cases). The SIRs of parental concordant (same site) tumours after prostate cancer were also increased for urinary bladder cancer (4.42, 4 cases) and chronic lymphoid leukaemia (38.0, 2 cases).

**Conclusion:**

A higher than additive and multiplicative interaction was observed between the individual history of prostate cancer and parental history of colorectal and urinary bladder cancers, although the number of cases did not permit the rejection of any interaction model. The results suggest that the occurrence of second tumours, for example bladder after prostate or prostate after bladder tumours, is mostly related to shared genetic and non-genetic risk factors rather than treatment of first cancer.

Prostate cancer is the most common cancer among males in Sweden, with an incidence rate up to 214 per 100 000 person-years (Centre for Epidemiology, 2006). The incidence of prostate cancer in Sweden has experienced an annual increase of 3.7% during the last decade. With the improvement in survival from prostate cancer, more and more survivors might face the problem of having an increased risk for subsequent primary tumours (Travis, 2006; Travis *et al*, 2006). An increased risk of subsequent prostate cancer after other types of cancer has been also observed (Dong and Hemminki, 2001; Diener-West *et al*, 2005; Sørensen *et al*, 2005; Neuzillet *et al*, 2007). For example, the within-patient clustering of bladder and prostate tumours has been extensively explored (Chun, 1997; Kouriefs *et al*, 2005; Liauw *et al*, 2006; Bostrom and Soloway, 2007; Kellen *et al*, 2007; Bostrom *et al*, 2008; Singh *et al*, 2008). The plausible aetiology of multiple subsequent tumours could be the effect of therapy, genetic and non-genetic risk factors, or the interactions between them (Hemminki and Boffetta, 2004; Travis, 2006; Travis *et al*, 2006). Aetiological studies have mostly focused on therapy-related factors, particular radiotherapy (Brenner, 2006; Kendal *et al*, 2006; Subramanian *et al*, 2007; Bostrom and Soloway, 2007). However, radiotherapy does not completely explain the increased risk of second tumours and limited information is available on the potential effect of family history on the risk of subsequent tumours after prostate cancer (Singh *et al*, 2008).

This study investigates the role of parental history in the development of second malignancies after prostate cancer, and the risk of subsequent prostate cancer in familial cases using the 2006 update of the nationwide Swedish Family-Cancer Database. Although cancer is mainly induced by environmental factors and only a small proportion can be explained by established genes, population-based estimation of parental risks is still a good measure of the familial clustering of tumours and reflects the sum of genetic risk effects attributable to both known and unknown genes.

## Materials and methods

The nationwide Swedish Family-Cancer Database includes offspring born in Sweden since 1932 and also their biological parents, with information about the first and second primary malignancies they experienced. Cancer was recorded according to the 7th version of the ICD code (ICD-7), and verified by the cytological or histological code. The agreement between clinical and cytological or histological diagnosis is close to 100% (Centre for Epidemiology, 2006). Unfortunately, information about cancer treatment is not included in the Swedish Cancer Registry.

Parental risks were investigated when fathers were affected by prostate tumour and when parents were affected by concordant (same site) cancer. On the basis of the Swedish Family-Cancer Database, the relative risk of second tumours in the general population and among individuals with a family history was estimated by standardised incidence ratios (SIRs), calculated as the ratio of the observed (*O*) to expected (*E*) number of cases. The follow-up started at diagnosis of prostate cancer and ended at the occurrence of death, emigration, the diagnosis of any subsequent malignancy or on 31 December 2004 (end of the study). Cases and person-years were classified according to the categories age (5-year groups), region (4 regions), calendar period (9 intervals) and socioeconomic status (6 groups). Confidence intervals (95% CI) were calculated assuming a Poisson distribution of the observed number of cases. To evaluate the risk of a second prostate cancer after any cancer, follow-up began at the date of diagnosis of the first malignancy and the uncensored event was the diagnosis of prostate cancer. Multiplicative interaction indexes (MIIs) and interaction contrast ratios (ICRs) were used to investigate the possible interaction between ‘individual history of prostate’ and ‘parental history of cancer’. If SIR_prostate_ represents the relative risk of cancer after prostate cancer, SIR_fh_ represents the relative risk among individuals with a parental history and SIR_prostatexfh_ represents the relative risk in prostate cancer patients with a parental history, MII=SIR_prostatexfh_/(SIR_prostate_ × SIR_fh_) and ICR=SIR_prostatexfh_−SIR_prostate_−SIR_fh_+1. MII≠1 indicates a departure from multiplicativity and ICR≠0 suggests a departure from additivity. Confidence intervals and *P*-values for MII and ICR were calculated by bootstrapping based on 100 000 replications. All analyses were carried out in SAS 9.1, the SAS Institute, Cary, NC, USA.

## Results

A total of 3 818 429 male offspring were registered and 18 207 men were diagnosed with prostate cancer from 1978 to 2004 in Sweden. Among them, 560 experienced a second malignancy after diagnosis; 75 269 men were diagnosed with any tumour other than prostate cancer and 838 of them had a subsequent prostate cancer.

The relative risk of cancer after prostate cancer is shown in Table 1 for sites with at least 11 observed subsequent cases (the same sites are shown in Table 2). Compared with the general Swedish population, prostate cancer patients showed an increased risk of cancer in the kidney (SIR: 1.75), urinary bladder (1.83) and skin (squamous cell, 1.67). The increased risk of colorectal cancer (1.23) showed borderline significance. Among patients with a parental history of prostate cancer, the risk was increased for bladder (2.44) and skin squamous cell cancers (3.34) and for myeloma (3.85). The third column of Table 1 shows the relative risks of second cancer in prostate cancer patients with a parental family history of concordant (same site) cancer. Prostate cancer patients with a parental history of colorectal cancer showed a 2.23 times higher risk of colorectal cancer than did men in the general population. Increased familial risks were also observed for urinary bladder (SIR: 4.42) and chronic lymphoid leukaemia (38.0, 2 familial cases affected after prostate cancer). As a control, the parental risk of these cancers as first primary was also shown in the fourth column.

Table 2 provides the risk of prostate cancer as second tumour. Increased risks were detected for kidney (1.56) and urinary bladder (2.25) cancer and for melanoma (1.22). In patients with a paternal history of prostate cancer, a significantly increased risk was observed for colorectal (1.88), renal (3.92) and urinary bladder (3.97) cancers. Among patients with a family history of concordant cancer, only by urinary bladder cancer showed a significantly increased risk after prostate cancer. It is important to point out here that urinary bladder cancer always showed a positive association with prostate cancer, both as first and as second primary tumour, both in the general population and among individuals with a family history.

Table 3 explores the interaction between the individual history of prostate cancer and family history of concordant cancer. For colorectal, urinary bladder and skin cancers, the interaction seemed to be higher than additive and higher than multiplicative. By contrast, a lower than additive and lower than multiplicative interaction was found for kidney cancer. However, the number of cases was small and it did not permit the rejection of any interaction model at the 5% confidence level.

## Discussion

This study focused on the effect of parental history of cancer on the development of subsequent tumours after prostate cancer, and on the risk of prostate cancer in cancer patients.

The Swedish Family-Cancer Database relies on national registries of complete coverage, thus minimising biases due to recall and ascertainment (Hemminki *et al*, 2001, 2006). Parents are registered at the time of birth of the child, thus allowing tracking of ‘biological’ parents in spite of divorce and remarriage. The study was limited because the maximum age in the offspring generation was 72 years, still below the highest risk for most cancer types, thus resulting in small numbers of familial cases. Moreover, no individual information on treatment or other potential cancer risk factors was available.

In this study, prostate cancer patients showed an increased risk of second tumours in the urinary bladder, kidney and skin (squamous cell). Patients affected by kidney and urinary bladder cancers, and by melanoma, showed an increased risk of subsequent prostate cancer. Novel data were related to the risk of second tumours in prostate cancer patients with a parental history of same-site tumours, which showed increased risks of colorectal (SIR=2.26) and urinary bladder (SIR=4.42) cancers and of chronic lymphoid leukaemia (SIR=38.0).

Cancer treatment with radiation and chemotherapy has been related to the development of second malignancies. Since 1980, surgery, hormonal therapy and radiotherapy have been widely used to treat prostate cancer. Chemotherapy has not been applied. It has been shown that prostate cancer patients undergoing external beam radiation therapy have a higher risk of secondary cancer than do surgically treated patients and patients treated with other forms of radiation (Neugut *et al*, 1997; Brenner, 2000, 2006; Baxter *et al*, 2005; Brenner and Hall, 2006; Moon *et al*, 2006). The potential contribution of radiotherapy to the development of second tumours should be reflected in an increasing risk with increasing time after first diagnosis. However, additional analyses of the database revealed decreasing risks with lead time between the two diagnoses, which would limit the role of radiotherapy in the development of subsequent tumours (data not shown).

In this study, 15–20% of patients with second urinary bladder or prostate cancer had a concordant parental history. The familial risk of second bladder cancer after prostate cancer (4.42) was higher than the relative risk of bladder cancer after prostate cancer (1.83) and higher than the familial risk of bladder cancer (1.79). Theoretically, a family history of cancer may lead to a more intense screening of prostate cancer. Previous analyses from the database found no increased risk of prostate cancer shortly after a paternal prostate cancer diagnosis and a borderline risk (*P*=0.07) after diagnosis of a brother (Bermejo and Hemminki, 2005). The absence of increased familial risks shortly after first diagnosis suggests a contribution of genetic factors rather than intensified medical attention.

On the basis of the independent effects of individual history of prostate cancer and family history of urinary bladder cancer and assuming an additive interaction model, the ICR would be 4.42−1.83−1.79+1=1.80 (Table 3). Analogously, the results suggested a higher than multiplicative interaction between the individual history of prostate cancer and family history of bladder cancer, but the number of familial cases was small to rule out other interaction models. Hardly any susceptibility genes are known that would explain shared effects in prostate and bladder cancers.

For second chronic lymphoid leukaemia after prostate cancer, it is difficult to explain the increased risks for a concordant parental cancer history, as no germline mutation has been identified so far (Sellick *et al*, 2005). Another new finding was the increased risks for skin squamous cell carcinoma after prostate cancer, when the offspring had a parental history of prostate cancer. Moreover, the MII suggested a higher than multiplicative interaction between the individual history of prostate cancer and family history of skin squamous cell carcinoma.

A family history of prostate cancer seems to have an important role in developing a second prostate cancer after any cancer other than prostate cancer, with an estimated SIR of 1.20. In addition to the candidate genes suggested earlier, a large number of low-risk variants have been associated with prostate cancer in genome-wide association studies (Dong, 2006; Eeles *et al*, 2008; Gudmundsson *et al*, 2008; Thomas *et al*, 2008). The interaction between family history and identified polymorphisms on prostate cancer risk has been investigated recently (Zheng *et al*, 2008). However, the present results indicate the association among discordant cancer sites, and the effects of most recently identified variants on colorectal, bladder and renal cancer have not been investigated yet.

On the basis of the Swedish Family-Cancer database, our results suggest that prostate cancer patients with a family history of cancer have an increased risk of concordant tumours in the urinary bladder. The increased risk in both directions – bladder after prostate and prostate after bladder tumours – points to the contribution of shared genetic and non-genetic risk factors. Although the number of parental cases was small, the data suggest that family history also seems to have an effect on other types of concordant cancers.

## Figures and Tables

**Table 1:** Relative risks of second cancer after prostate tumours and familial risks of first primary cancer

	**Risk of second cancer after prostate cancer**															
	**Male offspring**				**Sons of men with prostate cancer**				**Sons of patients affected by concordant cancer**				**Familial risk of first primary concordant cancer**			
**Type of second cancer**	***N***	**SIR**	**(95% CI)**		***N***	**SIR**	**(95% CI)**		***N***	**SIR**	**(95% CI)**		***N***	**SIR**	**(95% CI)**	
Upper aerodigestive tract	12	0.88	0.45	1.54	2	0.96	0.09	3.54	1	10.16	0.00	58.2	49	**1.67**	1.26	2.22
Stomach	18	1.23	0.73	1.95	1	0.47	0.00	2.70	0				131	**1.67**	1.40	1.99
Colorectum	92	1.23	0.99	1.51	6	0.54	0.20	1.19	11	**2.26**	1.12	4.06	1573	**1.69**	1.61	1.78
Pancreas	17	1.24	0.72	1.99	1	0.49	0.00	2.82	0				85	**2.01**	1.62	2.50
Lung	63	1.15	0.88	1.47	6	0.74	0.27	1.63	2	1.15	0.11	4.21	647	**1.72**	1.58	1.86
Prostate	18	0.05	0.03	0.09	4	0.08	0.02	0.22	4	0.08	0.02	0.22	2472	**1.88**	1.80	1.96
Kidney	33	**1.75**	1.20	2.46	2	0.71	0.07	2.61	1	2.72	0.00	15.6	119	**2.19**	1.82	2.62
Urinary bladder	76	**1.83**	1.44	2.30	15	**2.44**	1.36	4.03	4	**4.42**	1.15	11.4	279	**1.79**	1.58	2.01
Melanoma	24	0.93	0.59	1.38	5	1.22	0.39	2.87	0				438	**2.44**	2.22	2.68
Skin, squamous cell	37	**1.67**	1.18	2.31	11	**3.34**	1.66	6.00	2	4.42	0.42	16.3	172	**2.03**	1.74	2.36
Nervous system	25	1.47	0.95	2.17	3	1.14	0.22	3.39	0				257	**1.69**	1.50	1.92
Non-Hodgkin's lymphoma	33	1.44	0.99	2.03	2	0.58	0.05	2.13	1	3.24	0.00	18.6	167	**1.86**	1.60	2.17
Myeloma	14	1.61	0.88	2.71	5	**3.85**	1.22	9.06	1	11.7	0.00	67.0	35	**2.43**	1.74	3.40
Leukaemia	16	0.88	0.50	1.44	3	1.11	0.21	3.28	2	6.80	0.64	25.0	150	**1.87**	1.58	2.19
Chronic lymphoid leukaemia	8	0.97	0.43	1.93	3	2.46	0.46	7.29	2	**38.0**	3.58	140	46	**6.11**	4.47	8.15
Any type	560	0.72	0.66	0.78	79	0.69	0.54	0.86	305	0.80	0.69	0.87	81 548	**1.05**	1.05	1.06

Abbreviations: CI=confidence interval; *N*=number of individuals with second neoplasm; SIR=standardised incidence ratio. Bold refers to a significant risk increase with 5% statistical significance.

**Table 2:** Relative risks of prostate tumours as second cancer in the general population and among individuals with a family history

	**Risk of second prostate cancer**											
	**Male offspring**				**Sons of men with prostate cancer**				**Sons of patients affected by concordant cancer**			
**Type of first primary cancer**	***N***	**SIR**	**(95% CI)**		***N***	**SIR**	**(95% CI)**		***N***	**SIR**	**(95% CI)**	
Upper aerodigestive tract	24	0.84	0.54	1.26	6	2.64	0.95	5.79	0			
Stomach	14	1.00	0.54	1.68	2	2.19	0.21	8.05	1	1.26	0.00	7.24
Colorectum	92	0.80	0.65	0.99	16	**1.88**	1.07	3.07	6	0.49	0.18	1.08
Pancreas	2	0.32	0.03	1.17	0				0			
Lung	19	0.52	0.31	0.91	2	0.76	0.07	2.78	2	0.89	0.08	2.29
Prostate	18	0.05	0.03	0.09	4	0.08	0.02	0.22	4	0.08	0.02	0.22
Kidney	56	**1.56**	1.18	2.03	14	**3.92**	2.14	6.60	1	1.14	0.00	6.53
Urinary bladder	203	**2.25**	1.95	2.58	30	**3.97**	2.68	5.67	11	**2.85**	1.42	5.12
Melanoma	111	**1.22**	1.01	1.47	11	1.57	0.78	2.82	3	1.51	0.29	4.48
Skin, squamous cell	45	1.08	0.79	1.45	7	2.04	0.81	4.23	2	0.98	0.90	3.61
Nervous system	44	1.15	0.83	1.54	3	0.92	0.17	2.72	0			
Non-Hodgkin's lymphoma	35	0.74	0.52	1.03	5	1.33	0.42	3.14	1	0.70	0.00	4.00
Myeloma	8	0.63	0.27	1.25	2	1.92	0.18	7.04	0			
Leukaemia	24	0.79	0.51	1.18	4	1.70	0.44	4.41	1	1.05	0.00	6.01
Chronic lymphoid leukaemia	9	0.61	0.28	1.17	1	1.06	0.00	6.06	0			
Any type	856	0.79	0.74	0.85	129	**1.20**	1.00	1.42	471	0.89	0.81	1.00

Abbreviations: CI=confidence interval; *N*=number of individuals with second neoplasm; SIR=standardised incidence ratio. Bold refers to a significant risk increase with 5% statistical significance.

**Table 3:** Interaction between individual history of prostate cancer and family history of concordant cancer

													**Type of interaction**							
	**Individual history**				**Family history**				**Individual and family history**				**Additive**				**Multiplicative**			
**Type of second cancer**	***N***	**SIR**	**(95% CI)**		***N***	**SIR**	**(95% CI)**		***N***	**SIR**	**(95% CI)**		**ICR**	**(95% CI)**		***P*-value**	**MII**	**(95% CI)**		***P*-value**
Colorectum	92	1.23	0.99	1.51	1573	**1.69**	1.61	1.78	11	**2.26**	1.12	4.06	0.34	−0.78	2.40	0.68	1.09	0.55	2.15	0.59
Kidney	33	**1.75**	1.20	2.46	119	**2.19**	1.82	2.62	1	2.72	0.00	15.6	−0.27	−3.22	104	0.48	0.70	0.02	28.8	0.43
Urinary bladder	76	**1.83**	1.44	2.30	279	**1.79**	1.58	2.01	4	**4.42**	1.15	11.4	1.80	−1.28	11.2	0.81	1.35	0.42	4.35	0.69
Skin, squamous cell	37	**1.67**	1.18	2.31	172	**2.03**	1.74	2.36	2	4.42	0.42	16.3	1.70	−2.12	24.9	0.70	1.30	0.20	8.52	0.61

Abbreviations: CI=confidence interval; ICR= interaction contrast Ratio; MII=multiplicative interaction index; *N*=number of individuals with second neoplasm; *P*=probability value for ICR larger than 0 and MII larger than 1; SIR=standardised incidence ratio. Bold refers to a significant risk increase with 5% statistical significance.
